# Challenges to the Development and Implementation of Public Policies to Achieve Animal Welfare Outcomes

**DOI:** 10.3390/ani1010069

**Published:** 2010-12-31

**Authors:** Margaret Rose

**Affiliations:** Prince of Wales Clinical School, University of New South Wales, Sydney Australia; E-Mails: m.rose@unsw.edu.au; margaret.rose@sesiahs.health.nsw.gov.au; Tel.: +61-2-9382-3908; Fax: +61-2-9382-4049

**Keywords:** public policy, animal ethics committees, legislation, animal welfare outcomes, animal research

## Abstract

**Simple Summary:**

Many countries have enacted legislation to protect animals. In the 1800's the primary concern was to protect animals from cruelty but more recent legislative changes also seek to ensure that human beings uphold a duty of care towards those animals for which they are responsible. Today animal welfare concerns all aspects of our interaction with other animals. Although, the diversity of views in society can present challenges, the whole community needs to be engaged in the development and implementation of policies and initiatives so as to achieve sustainable improvements in animal welfare.

**Abstract:**

Although there is a long-established tradition of concern for the welfare of animals, it was not until the mid 1800's that governments sought to enact legislation to protect animals from cruelty. In the 1950's, questions concerning animal welfare re-emerged and in the ensuing years have been an on-going focus of government activities. These developments occurred against a backdrop of significant social change but there are important differences in what now underpins and informs these considerations. In the formulation and implementation of public policies, governments look for a course of action that represents and protects the interests of the community; the process may be challenging with competing interests but the final determination seeks a middle ground that best meets the needs and interests of the community as a whole. When policy development concerns our relationship with other animals, the complexity of this relationship presents particular challenges not only to the formulation of policies but also to the evaluation of outcomes. Notably, the depth of feelings and diversity of views in our community reflect the complex social, cultural and personal dimensions of this relationship. The use of animals for scientific purposes remains one of the most contentious animal welfare issues primarily because when animals are used for these purposes, accepted animal welfare benchmarks cannot always be met. Based on the Australian experience, this paper will discuss the influences in and on-going challenges to the development and implementation of public policy when animals are used for these purposes.

## 1. Introduction

We have only to look at the scope of topics covered at this conference to be reminded of the depth, diversity, richness and complexity of the relationships between human beings and other animals. Further, the central theme—“*Minding Animals*”—reminds us not only of our responsibilities and duties towards other animals but also of the challenges which confront us as we seek to understand the ability of animals to think and to feel and the pursuant ethical implications for us in our treatment of those animals. These are important drivers in the development of public policies concerning animal welfare that are shaped by our social and cultural values and beliefs, motivated by our desire to protect animals and informed by our understanding of the experiences of animals.

There is a long-established tradition of concern for the welfare of animals in many societies albeit reflecting differing cultural values and beliefs. In the 19th century, responding to concerns about cruelty towards animals, governments sought to enact legislation to prevent such practices and since that time have increasingly become involved in the development of policies, including legislation, regulations, codes of practice and guidelines relating to various aspects of the ways in which humans interact with other animals.

Such government intervention reflects both an increase in community interests in our duties and responsibilities towards other animals and identification of the need for government involvement in particular seeking to resolve the claims of competing community interests. These developments have occurred against a backdrop of significant social change; there are important differences in what now underpins concerns about animal welfare to the situation in the 19th century.

From the mid 1950's we have seen significant social and political forces that have shaped present government policies on animal welfare. First, and probably of greatest importance, has been the shift in our attitude towards other animals; the concerns of the 19th century, where protection from cruelty was the primary focus, have expanded to a broader platform. It now is recognized that there is a moral dimension to our considerations about how we treat other animals and, further, that our duties towards other animals should encompass not only protection from cruelty but also broader issues often discussed under the notion of ‘quality of life’ or described under the broad categories of the ‘Five Freedoms’—a shift that is exemplified in the goals of the Australian Animal Welfare Strategy [1]. Furthermore, when considering the ethical questions involved, we do not have a single view as to the value and claims of other animals; a range of perspectives, often evident as strongly held and polarized views, is seen in the community. In the development and implementation of policies, governments look for a course of action that represents and protects the interests of the community as a whole, taking into account competing interests and seeking a middle ground that best meets the needs of all stakeholders. Thus a lack of consensus as to the value and claims of other animals presents particular difficulties in the formulation of effective public policies.

Controversy about the use of animals for scientific purposes, be that research, teaching or product testing has been at the forefront of the animal welfare debate from the beginning of the modern animal welfare movement in the 19th century notably being one area where government intervention has been advocated. Such questions arise primarily because when animals are used for scientific purposes accepted animal welfare benchmarks cannot always be met; in limited circumstances, animals may experience discomfort, disease, pain or distress as a deliberate component of an experimental study. Clearly there needs to be special justification for this to occur—we need to decide when and if this is acceptable and, if so, by what criteria. Thus, ethical decisions have been important to our use of animals in science for many years. Moreover, it is recognized that these are questions that go beyond the purview of the scientific community; the wider community should be involved in these determinations.

This paper will describe how these matters have been addressed in Australia and, based on that experience, discuss the challenges to the development and implementation of public policy when animals are used for scientific purposes.

## 2. Setting Public Policy Goals

In Australia, in 1984, the Federal Government initiated a national enquiry into animal welfare by a Senate Select Committee. The outcomes of that inquiry, which was conducted over five years, were to set the benchmarks for the subsequent development of animal welfare policy in this country and continue to influence more recent developments such as the Australian Animal Welfare Strategy [1].

Animal experimentation was one of the major terms of reference for this enquiry with the *Report on Animal Experimentation* released in 1989 [2]. This Report identified the need to ensure that the use of animals is justified through a process of ethical review in which the principles of the 3Rs are applied—Replacing the use of animals where possible, and, when this is not so, to use the minimum number (Reduction) with minimum impact on their welfare (Refinement). The Senate Committee concluded that there was conditional community support for the use of animals in science but that it was paramount that the welfare of animals was promoted and protected; transparency of process, public participation in the ethical decision-making as well as mechanisms for accountability were key matters that needed to be addressed to enable public confidence that these goals were achieved.

The Senate Committee identified Animal Ethics Committees (AECs) as the ‘lynch pin’ in any system of ethical review arguing that it was the collective wisdom of a committee with each member bringing their own expertise and values to bear on a matter that would enable broadly based, collective judgments to be made. The Committee concluded that AECs served a major social benefit by bringing together people of differing views and saw a pivotal role for lay/non-scientist members reasoning that their involvement would improve the review process by providing a broader consideration of animal welfare issues. It was recognized that there was a need to reconcile human needs and animal interests and that such decisions needed to be informed by an examination of the evidence. Further, by providing an interface between scientists and the wider community, it was seen that the AEC could increase the awareness among scientists of wider community views and act as a vehicle for public accountability.

## 3. The Australian Code of Practice

The Senate Committee endorsed the *Australian Code of Practice for the Care and Use of Animals for Scientific Purposes* (the Code) as the national policy statement on the use of animals for these purposes—research, teaching and product testing.

The Code [3] defines the ethical framework for making decisions as to if and how animals can be used for these purposes in this Australia. The Code sets out the general principles that guide these decisions and details processes for ethical review, approval and monitoring. The Code encompasses all aspects of the care and use of animals involved in research, product testing or teaching activities in the fields of medicine, biology, agriculture, veterinary and other animal sciences. The Code concerns the use of all live, non-human vertebrates and higher order invertebrates such as cephalopods.

The Code was first published in 1969 as an initiative of the scientific community and since then has been revised on six occasions, most recently in 2004. An overview of the scope and contents of successive editions of the Code highlights how the key elements of the ethical framework have evolved [4,5].

The intent of the Code, as stated in the first edition, was to set down guidelines for the conduct of experiments involving animals to ensure pain or discomfort was prevented or minimized; in so doing, to promote humane conduct of these activities. The 1969 edition highlighted the scientific rationale for inferring that some animals could experience pain and the need to recognize that not only the experience of pain but of suffering, discomfort and distress also needed to be taken into account. Notably, the important link between animal welfare and scientific outcomes was highlighted—“*For humane reasons and to ensure minimum variability in experimental methods all laboratory animals should receive every consideration for their comfort and well-being*”.

The second edition of the Code in 1978 continued these themes. The underlying principle being that “*the lives of animals, especially vertebrates, should be treated with respect and care and their welfare catered for at all times*”; experiments involving animals needed to be justified and use the minimum number of animals. Of note, the Code now required institutions to establish an Animal Experimentation Ethics Review Committee to ensure investigations using animals conformed to accepted standards of animal care as set out in the Code.

During the 1980's there were three revisions of the Code in 1985, 1987 and 1990 with the fifth edition in 1990 being an important milestone. This edition brought forward the principles which had been developed in previous editions and detailed a framework for ethical review aligned with the goals set out in the Senate Committee Report and published international standards. This framework has withstood the test of time and has been substantially unchanged in subsequent editions of the Code.

The major addition to the Code in the sixth edition in 1997 was a section on wildlife research—an important development and one of the first national policy documents to address these issues.

The most recent edition of the Code in 2004, brought several important developments. A statement on the ‘duty of care’ of all those involved in the care and use of animals for research or teaching, although inferred in previous editions, was a timely clarification which serves to highlight this core value. The inclusion of definitions of pain, distress and well-being provides a broad and practical conceptual framework within which the impact of circumstances and procedures on the welfare of animals can be assessed and managed. Further, the inclusion of a requirement for external triennial review of an institution's ethical review procedures is a significant development as an additional mechanism to support institutional arrangements and accountabilities.

It is notable that throughout its history of over forty years, the foundation principles of the Code have been constant—respect for animals and a commitment to their welfare, the imperative to only use animals when justified, to promote the 3Rs and to recognize that inextricable link between animal welfare and scientific outcomes.

The Code is sponsored by the peak national organisations with responsibilities for the funding, or conduct, of scientific activities in Australia. From the first edition, the National Health and Medical Research Council (NHMRC) has taken a leading role in the development of the Code having been a sponsor since its inception and supporting its on-going revision and publication. The Commonwealth Scientific and Industrial Research Organisation (CSIRO) has endorsed the Code since the second edition and continues to do so along with the Australian Research Council (ARC) and Universities Australia (formerly the Australian Vice-chancellor's Committee) both of which have endorsed recent editions.

The capacity of the Code to deliver its stated goals rests on two key platforms—an ethical framework and effective governance arrangements.

## 4. An Ethical Framework

The fundamentals of the ethical framework that is established by the Code are a set of guiding principles that inform a process for ethical review; this review being undertaken by an Animal Ethics Committee (AEC) that must include representatives of the wider community in its membership.

Such a framework is important to facilitate effective ethical review. Although we can agree that our use of animals in these circumstances poses ethical questions that must be addressed, we do not have consensus as to the value we place on the interests of the animals when deciding if such use is acceptable. Consequently, a robust process that supports the expression of differing views, facilitates genuine debate and seeks to reach a conclusion through open and critical examination of argument, is important for there to be confidence in and broad acceptance of the outcomes.

The Code is not a prescriptive document but rather sets out principles which establish the criteria against which, on a case by case basis, the evidence put forward to support a decision to use animals can be tested. This approach permits a set of common principles to be applied to a wide range of circumstances. It provides flexibility and allows an in depth review of the specifics of a particular project so that the potential risks to the animals involved are identified and strategies to manage those risks are developed. Not only is this approach compatible with how science is done—how research is planned, reviewed and conducted—but, importantly, it promotes an assessment of animal welfare which is focused on the particulars of a project and supports the incorporation of new knowledge relevant to promoting animal welfare on an on-going basis. Further, based on statements of principle, the Code provides criteria to assess animal welfare outcomes for particular procedures, for example, in the choice of methods for anesthesia or euthanasia that are best suited to the species and the circumstances of the proposed study. Thus, a principle-based approach enables animal welfare outcomes in the context of scientific activities that would not be possible using a more prescriptive document.

Under the Code, institutions which use animals for scientific purposes must appoint an AEC whose primary role is to ensure that any use of animals within that institution is in accord with the principles of the Code; the AEC reviews and monitors all activities concerned with the breeding, supply and use of animals in these circumstances. Before approval is given for a specific project to commence, the Code requires AEC members to be satisfied that a case has been made that the proposal is justified taking into account the predicted scientific or educational benefit, the evidence that the use of animals is necessary and that, for those animals used, the study will involved the minimum number compatible with the scientific goals with minimum impact on their welfare *i.e.*, there is a critical evaluation that the principles of the 3Rs have been rigorously applied. Consequent AEC decisions should, where possible, be on the basis of consensus.

The membership and operation of the AEC are detailed in the Code that requires four categories of membership—a veterinarian with relevant expertise, an active scientist, a person representing animal welfare and a community representative.

Concerning the animal welfare member, the Code requires the appointment of ‘*a person with demonstrable commitment to and established experience in furthering the welfare of animals, who is not employed by or associated with the institution and not involved in the care and use of animals for scientific purposes*’. The community member also must be independent of the institution and not involved in the use of animals in scientific or teaching activities. It is intended that this person will bring to the AEC a wider, independent community view not reflected in the expertise and experiences of other categories of membership.

While there can be more than one person appointed to each membership category, if more than four members, the animal welfare and community members must represent at least a third of the total membership. Approval of projects can only occur at AEC meetings where attendance of members from each category is necessary for a quorum. The AEC Executive must have at least one animal welfare or community member.

AEC membership is drawn from a spectrum of interests, seeking to bring a diversity of views to its deliberations. There will be tensions between competing values and interests; differences that can place particular demands on AEC members. Whilst it is recognised that external members, in particular animal welfare and community members, play a critical role, their engagement with the AEC process can be problematic by virtue of their being external to the organization and, often, with limited scientific knowledge. The AEC process must be inclusive; all members must be involved. Members need to be informed of issues, aware of sensitivities and, when necessary, committed to the resolution of differences. The provision of complex technical information to members of AECs in lay language is an essential and sometimes challenging aspect of this process.

In the 1980's, there was considerable debate around two issues concerning the operation of AECs. The first concerned whether or not AECs should operate within, or be external to, institutions. The Senate Committee supported the argument that had been made in a number of submissions that institutional AECs would be more effective by the close supervision they could bring to the conduct of such research. As will be discussed below, the placement of the AEC within an institution is integral to the effective governance of these activities.

The second matter concerned the involvement of members of animal welfare organisations—on one hand concern by scientists that they could have a negative influence on the determinations of the AEC, on the other, concerns expressed by members of animal welfare organisations that their involvement in the AEC process was tantamount approval of activities with which they disagreed. Despite these misgivings, members of animal welfare organisations have proven to play a significant role on AECs.

The Code is a living document; to be relevant and influential it needs to respond to emerging community concerns and to reflect advances in scientific knowledge whether that be procedures which present specific ethical dilemmas or information concerning animal welfare. Further, as argued above, it is important that ethical decisions are informed by evidence in support of claims to justify the use of animals. Thus, over the course of Code revisions there have been changes in detail and content concerning specific matters and, in areas where more detailed information would be helpful, policies and guidelines have been developed to supplement the Code and to provide contemporary, evidence-based information as a resource for researchers, teachers and AEC members in their deliberations. For example, NHMRC publications such as the *Guidelines to Promote the Wellbeing of Animals Used for Scientific Purposes* [6,7] and the guidelines on GM animals [8,9] and the series of publications from the NSW Animal Research Review Panel of guidelines for the care and housing of a number of laboratory species, including rats, rabbits, mice, guinea pigs and sheep [10].

## 5. Governance

It is expected that research and teaching are conducted to high ethical and scientific standards [11]. Thus, institutions involved have in place mechanisms to be able to demonstrate that there is effective over-sight of these activities, and that standards of quality, safety, and ethical acceptability are being met. Not only are researchers, teachers and institutions responsible for meeting these standards but they need to be able to demonstrate that they are doing so. Notions of responsibility and accountability are central to effective governance.

The Code details the responsibilities of all involved in the care and use of animals when they are used for scientific purposes. The responsibilities of researchers and teachers, of institutions and of AEC members are clearly set out; the AEC processes for approval, monitoring and reporting provide mechanisms for accountability.

The primacy of the responsibility of the researcher (or teacher) is a cornerstone of the Code. The Code assigns to the researcher “*direct and ultimate responsibility for all matters relating to the welfare of animals they use*”. The Senate Committee in its Report on Animal Experimentation contended that assigning this responsibility to the researcher or teacher would achieve more for animal welfare that the most stringent monitoring or supervisory system—an argument that is endorsed by many commentators [12].

Notions of responsibility and accountability are complementary but the processes used to achieve accountability have implications for how responsibility is exercised. If accountability is sought through a system of control, this will devolve responsibility from the practitioner to the review authority and distance the practitioner from responsibility for his or her actions. When accountability is achieved through a process of authentication this will act to enhance the responsibility of the practitioner.

Thus there are significant implications for achieving the goals of the Code in relation to effective ethical review and animal welfare outcomes depending upon the governance arrangements [13]. The Code is clear as to the relationship between the AEC and researchers and teachers and their respective responsibilities; the role of the AEC is to verify the case to use animals and to monitor approved activities—it is a process of authentication which supports, but holds accountable, the actions of researchers and teachers and, in so doing, seeks to optimize achieving animal welfare outcomes.

## 6. Legislation

Legislative responsibility for animal welfare in Australia is vested in the States and Territories and, today, each have enacted legislation that regulates the use of animals in research and teaching. Although there are some differences, in all cases, the Code has been incorporated into legislation and so is the national policy that sets the standards for such use of animals [14]. Thus legislation provides a formal mechanism for institutions to be accountable to the wider community for the governance of these activities but, importantly, has been crafted to support the governance framework established under the Code; institutions and governments have complimentary responsibilities in the oversight of these activities.

In their consideration of the legislative control of animal experimentation, the Senate Committee supported separate legislation based on a duty of care and respect for animals. The Committee noted the benefits of an accreditation system, as opposed to a licensing process, and concluded that both institutions and government shared the responsibility to ensure that such activities were in accordance with agreed guidelines. They recommended a model of enforced self-regulation that calls upon the authority of both the State and the institution to effect compliance as being the best way to protect the welfare of animals used in science in Australia. The Senate Committee cautioned that there needed to be a balance between the activities of the State and of the institutions; this relationship should be complementary, not competitive. Further, in this legislative model, the Committee concluded that the incorporation of the Code into a legislative framework should be based on principles of good conduct rather than prescription of practices as the most effective way of promoting the 3Rs.

In a recent review of the legislative models that could be used to regulate animal welfare and so promote and protect improved animal welfare outcomes Bloom [15] concluded that the difference between the notions of animal welfare and prevention of cruelty towards animals necessitated different legal approaches and that confounding these objectives within the same legal process impeded successful outcomes in either circumstance. In supporting the need for different legislative processes, Bloom contended that legislative models designed to prevent cruelty operate within the framework of criminal law and that that premise was not suited to promotion of animal welfare that relied upon voluntary compliance within lawful and socially sanctioned use of animals. Accordingly, he recommended a shift in the regulation of animal welfare from a legal process aligned to criminal sanctions towards a social process where regulators and industry worked together to solve issues and promote improved animal welfare standards—a similar approach to that advocated by the Senate Committee.

In this model, the *Animal Research Act* was introduced into New South Wales in 1985. This legislation pioneered a new approach to considerations of animal welfare not only in Australia but internationally. This legislation seeks to provide a balance between accountability for decisions to use animals, the conduct of those activities and the benefits to the community from scientific enquiry. One of the basic tenets of this legislation is the need to recognize the differing views held in the community about animal experimentation in such a way as to bring a broadly-based consensus to decisions about how and why animals are used.

The *Animal Research Act* establishes a legal framework within which the Code operates, requiring that any use of animals for scientific purposes must be approved by an AEC in accord with the principles of the Code, and mandates public participation in both the decisions as to when and how animals are used for scientific purposes and the oversight of those activities. Thus, the involvement of and accountability to the wider community is a central platform of this legislation.

When introducing this legislation to Parliament, the Minister asserted the following key issues as foundations to this legislation. A duty of care was identified as a core value, with the investigator having primary responsibility for the welfare of animals involved in an approved project. The role of the institutional AEC was seen as pivotal as was the participation of the wider community. The legislation also recognized the dual responsibilities of institutions, through their AECs and of government to monitor animal welfare outcomes.

Government monitors compliance with this legislation and the principles and practices of the Code through the Animal Research Review Panel (ARRP); a statutory body of twelve with equal representation from research institutions, government and animal welfare organisations. Through its varied activities the ARRP seeks to protect and enhance the welfare of animals used in research, teaching and product testing and to promote within the community an appreciation of the ethical and scientific issues involved in that use.

One of the ARRP's major activities is to visit institutions to audit compliance with the legislation and the Code. These visits are conducted by a team of the ARRP members, if possible including an animal welfare representative, whose task it is to gather objective evidence of the effectiveness of the AEC, the adequate provision of animal care and the operation and management of facilities in accord with the principles of the Code [16]. The team reviews AEC membership, procedures, records, reports and monitoring, meets with AEC members and investigators and visits animal holding facilities and laboratories. These activities enable the ARRP to identify areas where additional policies and guidelines will assist in the implementation of the Act and the Code (for details refer www.animalethics.org.au).

The ARRP produces an Annual Report to Parliament which, *inter alia*, documents statistics on the use of animals and initiatives to achieve the 3Rs and thus reports on animal welfare outcomes.

## 7. Discussion

In Australia, these policies have evolved on the understanding that the processes and outcomes achieved will meet the expectations of both the scientific and the wider community in terms of the needs of science, of animals and of public accountability.

If there is to be public confidence that the policies are achieving the desired outcomes, such evidence must be in the public domain. To this end, there should be evidence of effective ethical review and public engagement in the processes for ethical review and monitoring as well as in revisions of public policies including legislation and the Code; animal welfare outcomes should be measured by evidence of implementation of the principles of the 3Rs.

### Public participation

It is inescapable that questions about our use of animals in science must involve the wider community. In Australia, this occurs at three levels—through AEC membership, through involvement in the external triennial review of AECs (or in the case in NSW through the ARRP) and through participation in the revisions of the Code [17].

Involvement with AECs, as outlined above, is an essential avenue for wider community participation in decisions as to why and how animals are used for scientific purposes. Consequently, evidence of effective public participation is relevant to the successful operation of an AEC.

The only study to date concerns the operation of AECs in Canada [18] and identified a number of factors that, the authors concluded, could lead to bias in the AEC's determinations. They cited a preponderance of institutional members and potentially an intimidating process from the perspective of a ‘minority’ member—non-institutional members possibly without scientific expertise or differing animal welfare views—as matters of concern. Similar issues have been identified in a number of studies of human research ethics committees [19]. A common theme in all these studies has been an identified need to clarify the role of community members. In a detailed consideration of these questions, Dyer [20] supported the conclusions of Schuppli and Fraser [18] of the need to better define and understand the role of lay members on ethical review committees so as to fully realize the potential value from their involvement.

The 2004 edition of the Code included details for the external review of the operation of institutional AECs; the aim being to validate that the welfare of animals used by the institution was ‘safeguarded in accordance with the Code’. Members of the review team must be external to the institution and may include animal welfare or lay members from other AECs. This review is seen as providing an opportunity for self-assessment by the institution with feedback from the review team bringing new, or different, perspectives and so assisting the institution to evaluate, and if necessary modify, its procedures. Given that, in the course of this review, the AEC process is assessed as to whether it is ‘fair and transparent’, the important issues identified in the Canadian study can be addressed and remedial action taken as necessary.

The review of the Code is conducted under the auspices of the NHMRC. Through the processes required under the NHMRC's Policy on Public Consultation [21], the Australian community is actively engaged in this process. The three most recent revisions (1990, 1997 and 2004) have involved community representation and consultation. In 1997, the Code Liaison Group (CLG) was established to have on-going responsibility for the revision and oversight of the Code. Membership of the CLG comprises nominees the Code sponsors together with representatives of the State and Territory government departments with legislative responsibilities, the Royal Society for the Prevention of Cruelty to Animals and Animals Australia, the latter being major national animal welfare organisations. The review takes into account both scientific and technical developments as well as community views about the ethics of animal use in research, teaching and product testing. All submissions to the review are taken into account in the drafting of the final document.

Analysis of submissions to the Code revisions is one indication of the level of involvement by interested parties. Data for 1990—2004 show that whereas animal welfare organisations represented only 6% of respondents in 1990, in 1997 this increased to 22% and was sustained at a comparable level (18%) in 2004. In the same time, the number of individual submissions fell from 12 to 6% and submissions from institutions varying from 69% in 1990 to 38% in 1997 and 66% in 2004. Thus there is evidence of sustained, active involvement by interested parties and on-going participation by the non-scientific community.

### Public accountability

Since 1991, the ARRP has published an Annual Report to Parliament. Matters covered include details of ARRP activities for the previous twelve (12) months, a list of institutions audited, specific examples of achievements in animal welfare, determinations on complaints investigated, matters which have been identified as requiring further attention, a report on both the outcomes of the operational plan for the previous year and future plans and directions and details of the use of animals by species, purpose and procedures involved. In addition, institutions are asked to report the number of AEC meetings held each year and examples of how the principles of the 3Rs have been implemented. Such data relate to activities in New South Wales only there being no comparable report required in other states. Nevertheless, NSW data indicate the kinds of issues common to all jurisdictions.

The ARRP annual statistics of animal use are derived from information provided by accredited institutions. Data cover not only the use of animals in laboratory-based research but also field trials, product testing, diagnosis, the production of biological products, environmental studies and tertiary, but not primary or secondary, teaching. Data are provided and analyzed on the basis of species and objectives, e.g., diagnostic, education, immunology, nutrition, toxicology *etc.*, and procedures, e.g., breeding and maintenance of stock, observational studies, minor or major surgery, minor or major physiological challenges, lethality testing and studies involving the production of genetically modified animals (details of ARRP Annual Reports available at www.animalethics.org.au).

However, if we consider these data in light of the expected outcomes of the AEC process—that the use of animals was justified and necessary, the minimum numbers of animals were used and pain and distress were minimized—it is not difficult to understand how confusion and indeed skepticism can arise about the effectiveness of this process to deliver expected outcomes; the frequent public debates about the interpretation of statistics of animal use bear witness to this misunderstanding. Unfortunately, there is no direct link between the outcomes of AEC deliberations and the cumulative data of animal use. These are two separate domains of information [22].

Data published in the annual statistics represent the absolute level of animal use; analysis of such data will indicate general patterns and trends. The outcomes of the AEC process are linked to and defined by the circumstances of each project. The statistics of animal use, as presented, will not record when animals have been replaced in a protocol nor when strategies have been employed to reduce the numbers of animals used. Data on the kinds of procedures to which animals are subjected will not reflect the specific strategies which may be employed to refine practices nor procedures to minimize that impact.

One way to achieve some indication of how data of animal use relates to the goals of the AEC process may be to consider these data against a background of general indicators of research activity, such as numbers of grants or related publications. Also, publication of case studies that reflect how this process has achieved expected outcomes may be helpful.

Unquestionably, the information provided in annual statistics of animal use must be in the public domain. Information about the numbers and species of animals involved and what happens to those animals are matters of public interest and concern [23]. However, it is equally important that it is understood that this kind of information does not indicate whether or not, for a specific project, the use of animals has been justified or that the 3Rs have been applied. This also is a matter of public concern and importance. The challenge is how this kind of information can be readily provided.

Notably, the ARRP annual report also contains other kinds of information that have some bearing on the outcomes of the AEC process and the general outcomes sought through the Code. Institutions are asked to provide details of how the 3Rs have been implemented. However, we know that, particularly when animals are replaced and therefore an application does not come before the AEC, there is no readily available record and, to date, such reports are limited.

### Effective ethical review

Given the pivotal role of the AEC, confidence in its processes to deliver outcomes is at the heart of acceptability of the policies we have developed in Australia. If the AEC is to be an effective conduit for institutions and individuals to be accountable to the wider community, the interpretation of the kinds of information in the public domain is a critical issue and hence an understanding of the limitations to these data is important.

The AEC process is both informed by and responsive to the breadth of perspectives that are brought to these deliberations. We should be mindful that a decision that a proposed use of animals is justified must ultimately reflect a value judgment. Animal experimentation, as indeed any scientific activity, operates within a *milieu* of social and cultural values and influences. The argument for making such decisions by a committee is the benefit of bringing a range of values and perspectives to the deliberations. But for each individual, the values that will influence or determine a decision are complex. One of the consequences of the dynamic of this decision-making process is that it is inevitable that there will be a level of inconsistency in the decisions of the AEC; circumstances that also may contribute to a view that this represents a failure of the ethical review process rather than evidence of a strength of the process.

## 8. Conclusions

In Australia, the reports of the Senate Select Committee in Animal Welfare established the footprint for the subsequent development of public policies in relation to animal welfare including, most recently, the Australian Animal Welfare Strategy [1]. These developments have occurred over a twenty five year timeframe during which the Code has been revised on three occasions and has been incorporated in various ways into State legislation.

The Code is closely aligned with the goals of the Australian Animal Welfare Strategy [1]—it underpins a national approach to ensure high ethical and scientific standards when animals are used in research and teaching; it involves the wider community in its development and implementation and, by responding to changing community views and incorporating scientific advances, it seeks to achieve sustainable improvements in animal welfare.

Under the Code, Australia has developed a strong ethical framework based on a system of ethics committees and supported by effective governance arrangements. These arrangements ensure both the involvement of and accountability to the wider community and, by placing primary responsibility for the welfare of animals used on researchers and teachers, seek to achieve the best possible outcomes for the welfare of those animals. Further, the *Animal Research Act* established a new approach to the incorporation of a code into legislation and provides a different legislative model compatible with achieving animal welfare outcomes and thus has wide applicability.

To be sustainable and to achieve policy goals these arrangements must be flexible and responsive. Key to this will be to ensure there are robust and effective procedures to identify and address barriers to participation and to review the currency of animal welfare standards so that decisions are based on contemporary scientific evidence and reflect community standards.
